# Noninvasive and portable stroke type discrimination and progress monitoring based on a multichannel microwave transmitting–receiving system

**DOI:** 10.1038/s41598-020-78647-x

**Published:** 2020-12-10

**Authors:** Jia Xu, Jingbo Chen, Wei Yu, Haisheng Zhang, Feng Wang, Wei Zhuang, Jun Yang, Zelin Bai, Lin Xu, Jian Sun, Gui Jin, Yongjian Nian, Mingxin Qin, Mingsheng Chen

**Affiliations:** 1 College of Biomedical Engineering, Third Military Medical University (Army Medical University), Chongqing, 400030 People’s Republic of China; 2 Institute of Brain and Intelligence, Third Military Medical University (Army Medical University), Chongqing, 400030 People’s Republic of China; 3 Department of Neurosurgery, Southwest Hospital, Chongqing, 400030 People’s Republic of China

**Keywords:** Cardiovascular diseases, Biomedical engineering

## Abstract

The hemorrhagic and the ischemic types of stroke have similar symptoms in the early stage, but their treatments are completely different. The timely and effective discrimination of the two types of stroke can considerable improve the patients' prognosis. In this paper, a 16-channel and noncontact microwave-based stroke detection system was proposed and demonstrated for the potential differentiation of the hemorrhagic and the ischemic stroke. In animal experiments, 10 rabbits were divided into two groups. One group consisted of five cerebral hemorrhage models, and the other group consisted of five cerebral ischemia models. The two groups were monitored by the system to obtain the Euclidean distance transform value of microwave scattering parameters caused by pathological changes in the brain. The support vector machine was used to identify the type and the severity of the stroke. Based on the experiment, a discrimination accuracy of 96% between hemorrhage and ischemia stroke was achieved. Furthermore, the potential of monitoring the progress of intracerebral hemorrhage or ischemia was evaluated. The discrimination of different degrees of intracerebral hemorrhage achieved 86.7% accuracy, and the discrimination of different severities of ischemia achieved 94% accuracy. Compared with that with multiple channels, the discrimination accuracy of the stroke severity with a single channel was only 50% for the intracerebral hemorrhage and ischemia stroke. The study showed that the microwave-based stroke detection system can effectively distinguish between the cerebral hemorrhage and the cerebral ischemia models. This system is very promising for the prehospital identification of the stroke type due to its low cost, noninvasiveness, and ease of operation.

## Introduction

The stroke is becoming increasingly prevalent worldwide. Commonly, the stroke can be divided into two types, namely, the hemorrhagic and the ischemic stroke, which have totally different treatments. The mismatched treatment leads to disastrous consequences, thereby making the diagnosis of the stroke type imperative before treatment^1^. According to the American Stroke Association and clinical thrombolysis guidelines, the first 4.5 h after ischemic stroke is “the golden period” for receiving the thrombolytic therapy^2,3^. However, if the thrombolytic therapy is unfortunately performed in patients with hemorrhagic stroke, the outcome would be severe. At present, only 10% of patients with ischemic stroke have received thrombolytic treatment during "the golden period"^4,5^. Therefore, a portable, rapid, easy-to-operate device is needed for the prehospital diagnosis of the stroke type and acute phase of stroke monitoring.

Recently, the mobile CT equipment has been used for the prehospital diagnosis of stroke^6,7^ and significantly shortens the time between the ischemic stroke attack and the thrombolytic therapy^5^, but its application is limited by the requirement of specialized medical vehicles, trained medical staff, and stable environment^7,8^. Additionally, the mobile CT equipment cannot be used to continuously monitor rapid pathologic lesions in the acute phase of stroke.

Electromagnetic-based devices, such as electrical impedance tomography^9,10^, magnetic induction technology^11^, and microwave technology^12^, show their advantage of noncontact and low cost for the prehospital diagnosis and monitoring of stroke. However, the clinical application of these electromagnetic-based methods is still in its infancy. The recently reported volumetric impedance phase shift (VIPS) is a magnetic induction device^11^ used for stroke detection. The VIPS can discern severe stroke from other subjects with a sensitivity of 93% but cannot diagnose the stroke type.

The microwave technology has been applied to brain function monitoring after several decades of development^12^ and has the potential to detect stroke type for the prehospital diagnosis by microwave image and microwave scattering parameters in a noncontact manner. The research activities of stroke microwave imaging focus on two directions, i.e., the design and the realization of imaging system^13–16^ and the development of inversion procedures for the dielectric imaging^17–21^. However, in contrast to the breast cancer detection^22,23^, the detected image quality of the brain is limited by the cerebral complex structure.

In addition to the microwave imaging, microwave scattering parameters are used in stroke diagnosis^24–31^. These studies focus on computer simulations^24,28,29^ or phantoms^25–27^ and rarely on in vivo experiments. In 2014, Persson et al. have performed a clinical study on 25 patients with acute stroke^31^. Their first-generation microwave system, Strokefinder R10 (Medfield Diagnostics AB, Gothenburg, Sweden), is used to differentiate the hemorrhagic and the ischemic stroke. Results show that patients with hemorrhagic stroke can be accurately identified at 90% detection sensitivity, but the classification results of ischemic patients are not satisfactory.

The simulation and the phantom studies have verified the theoretical feasibility of stroke diagnosis based on microwave scattering parameters, but this method cannot replace in vivo experiments due to many uncontrollable factors and individual differences among patients, such as age, gender, weight, stroke progress, site of lesion, and physical conditions. These factors affect the quantitative analysis of microwave-based stroke diagnosis. Therefore, the characteristics of microwave-based stroke detection with animal experiments in controlled conditions should be determined. A previous animal study^32^ has demonstrated that the hemorrhagic stroke can be effectively identified from nonstroke on the basis of our microwave scattering parameters stroke detection system under the selected measuring frequency band. However, further studies are still needed to differentiate the hemorrhagic and the ischemic stroke.

This study aims to validate the diagnosis and the monitoring of stroke types on a multichannel microwave-based stroke detection system without imaging by performing controlled experiments on an internal capsule cerebral hemorrhage model and a bilateral carotid artery ligation ischemia model in rabbits. Several improvements are taken on the stroke detection system to obtain a satisfactory accuracy of stroke classification. First, a new Euclidean distance transform method, namely, the reflection signal Euclidean distance (RSED), is proposed to transform the microwave scattering parameters into a new parameter, thereby improving the discrimination between the hemorrhagic and the ischemic stroke. Then, the transformed data are processed through the dimension reduction to decrease the redundancy from frequency and channel numbers. Lastly, the support vector machine (SVM) classification algorithm is used for the small sample classification.

In the “Results” section, the type and the severity of stroke are classified on the basis of the single-channel and multichannel RSED parameters. The spectrum results of the single-channel RSED of two stroke types and the SVM classification results obtained using the single-channel RSED are shown. The single-channel RSED has poor capability (50%) to distinguish the severity of stroke at low resolution (1 ml, 6 min). As such, the multichannel RSED is used for the SVM classification. The RSED parameters of the two stroke types obtained using the single-channel and the multichannel methods are compared, and the SVM classification is performed on the multichannel RSED parameters. Moreover, the effects of dimension reduction and kernel function on the SVM classification by using the multichannel RSED are demonstrated. The detailed research steps, such as RSED definition, microwave system structure, animal experiments, signal preprocessing, classification, and statistical methods, are introduced in the “Materials and methods” section.

## Results

**Figure 1:**
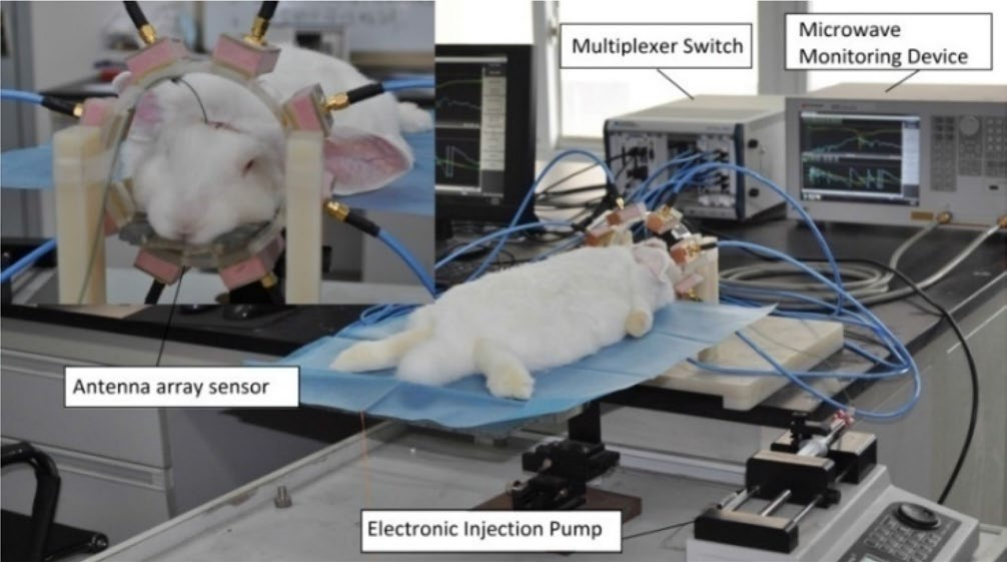
Experimental setups for monitoring cerebral hemorrhage in rabbits with the multichannel microwave-based stroke detection system.

Figure 1 shows the experimental system used in this study. The system consists of an antenna sensor array around the head of a subject, a microwave monitoring device, and a multiplexer switch. The measurement steps consist of monitoring the amplitude and the phase of the microwave scattering signal, conversion of RSED parameters, and evaluation of the postoperative changes in the bulk electromagnetic properties of the brain tissue. A detailed description of the system is given in the “Materials and methods” section.

### RSED single-channel detection by using channel 1-1

**Figure 2:**
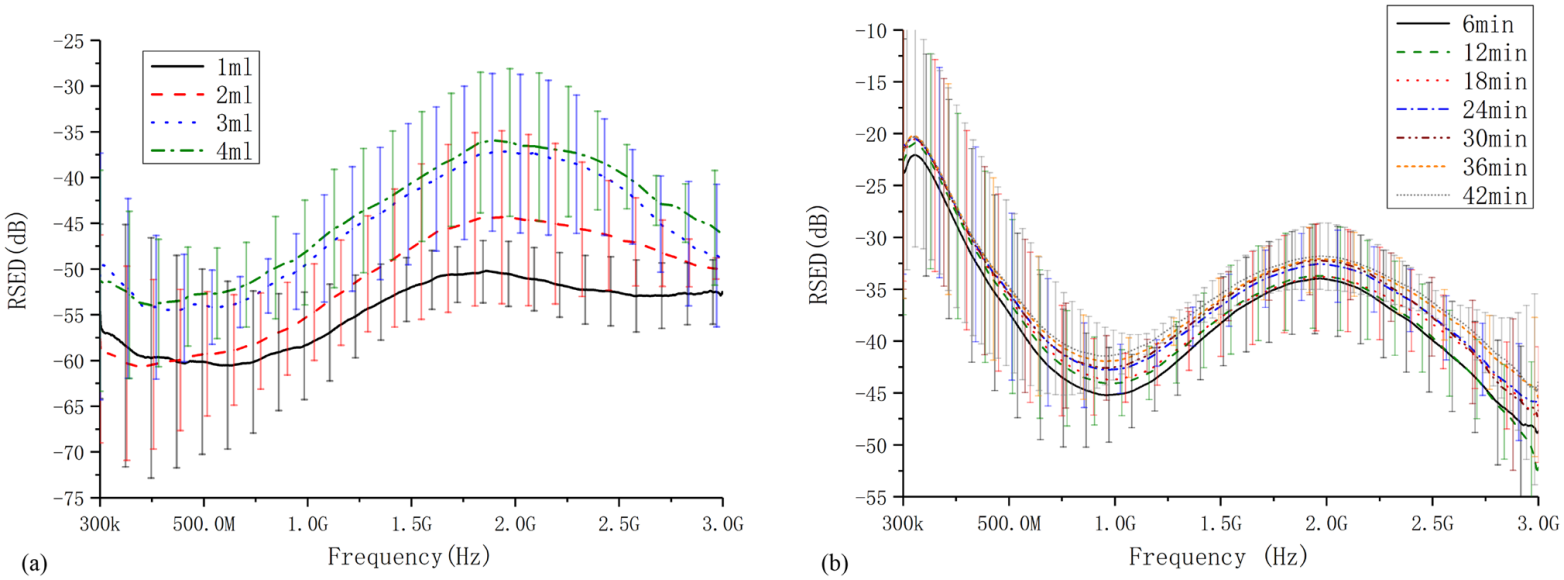
The average RSED of two groups (channel 1-1). (**a**) The RSED for the cerebral hemorrhage rabbit model. (**b**) The average RSED for the cerebral ischemia rabbit model.

The raw results of RSED in two groups (mean ± standard deviation [SD], channel 1-1) are shown in Fig. 2. Results show that the RSED for the cerebral hemorrhage increases with increasing bleeding volume (Fig. 2a) at frequency above 500 MHz. The RSED also gradually increases with increasing ischemic duration (Fig. 2b). At the same time, the detection sensitivity of the RSED for the cerebral ischemia is lower than that for the cerebral hemorrhage over the duration of the experiment. In the cerebral hemorrhage and the cerebral ischemia models, the RSED has a different correlation with frequency at different frequency ranges. The RSED increases in the band between 1 and 2 GHz of the two stroke models and decreases in the band above 2 GHz. The RSED of the hemorrhage model does not fluctuate too much with frequency below 1 GHz, whereas that of the ischemia model is reduced by at least 15 dB.

### Stroke diagnosis and monitoring with single-channel measurement

**Figure 3:**
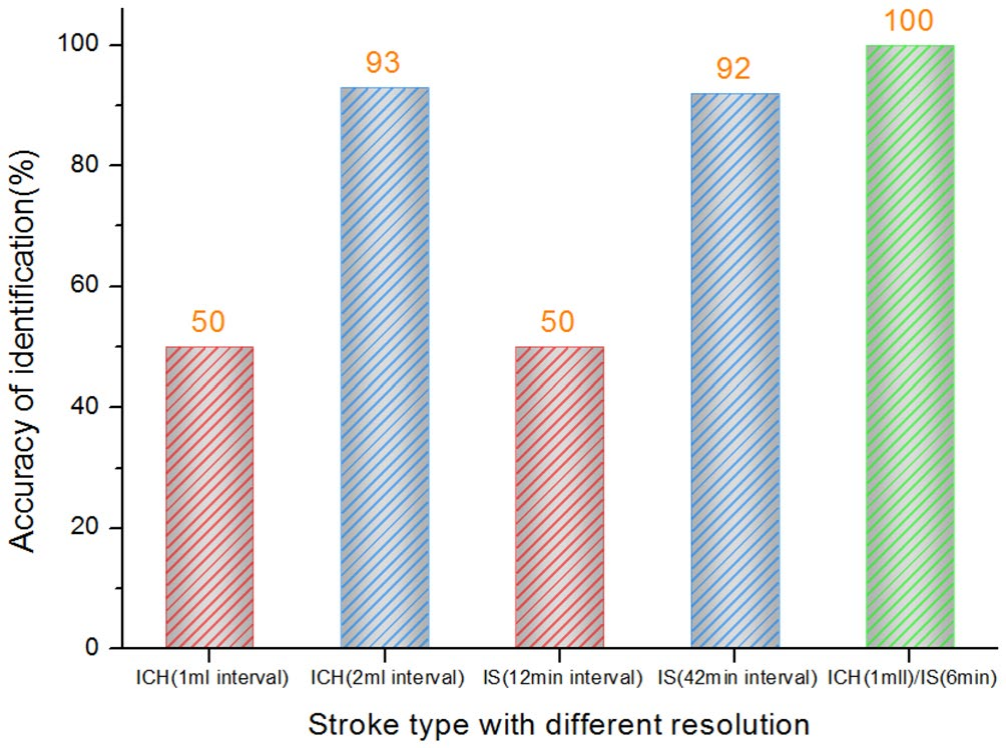
Identification results of cerebral hemorrhage and cerebral ischemia based on single-channel microwave detection (channel 1-1).

The discrimination between the hemorrhage and the ischemia stroke and the progress of hemorrhage or ischemia with different intervals are evaluated using the RSEDs of the single-channel (channel 1-1) measurement for classification. With the new RSED parameter, the identification of the stroke type can be done with high accuracy (reaching 100%), but the progress between the hemorrhages cannot be identified accurately. For the cerebral hemorrhage model, the accuracy of the 1 ml interval is 50%, and for the cerebral ischemia model, the accuracy of the 12-min interval is just 50% (Fig. 3). The single-channel microwave detection system can classify the stroke type but identifies the progression of either model with an unsatisfactory accuracy especially for mild pathological changes.

### Comparison of the RSEDs in single-channel and multichannel measurements

**Figure 4:**
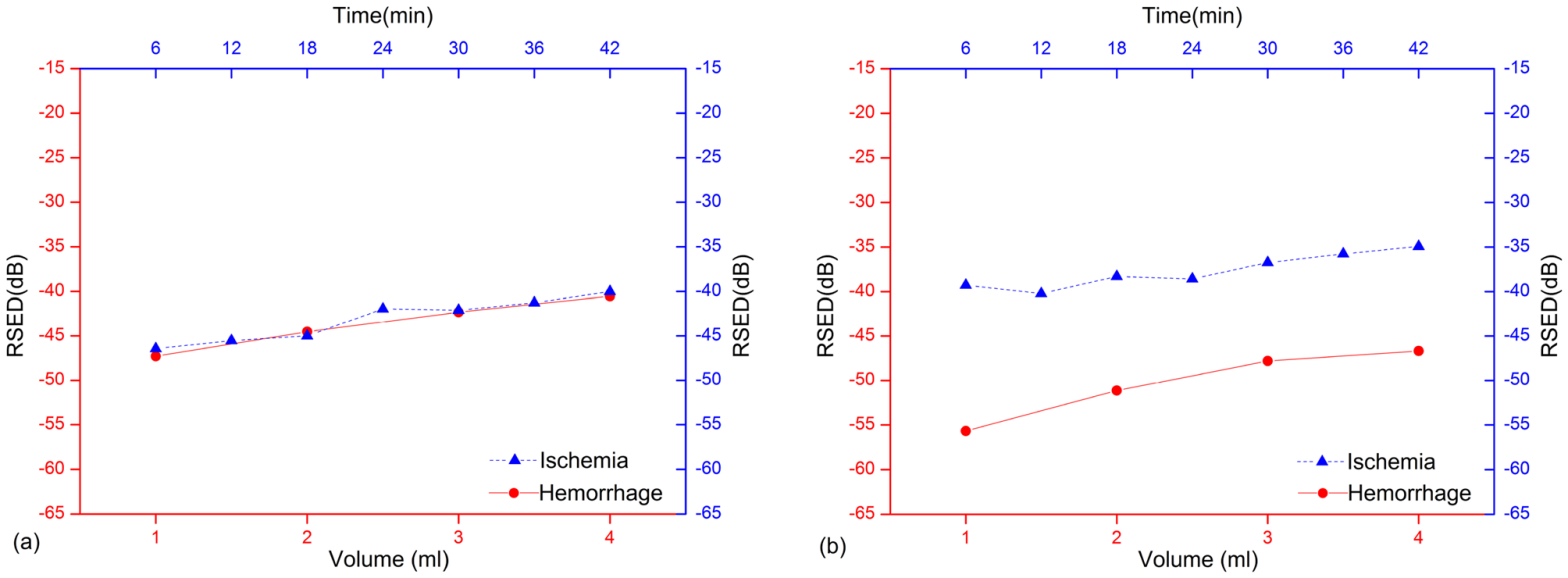
The RSEDs from single-channel measurements and 16-channel measurement in cerebral hemorrhage and cerebral ischemia (No. 4 and No. 6, 1.2 GHz). (**a**) The RSEDs from single-channel measurements (channel 1-1). (**b**) The RSEDs from the 16-channel measurement.

The channel 1-1 is selected from the multichannel array as a single channel and compared with the entire multichannel array to differentiate between multichannel and single-channel measurements. The difference in the RSEDs between the two stroke types in the multichannel detection is remarkably higher than that in the single-channel detection (by using sample 4 in the hemorrhage group and sample 6 in the ischemia group as an example at detection frequency of 1.2 GHz). The mean difference of the RSED between the two types of stroke with multichannel is 12.636 dB, comparing 0.483 dB with single-channel. Considering the trend of the data, the single-channel RSEDs for the hemorrhage and the ischemia groups are consistent and difficult to distinguish (Fig. 4a). The 16-channel measurement shows that the RSED calculated for the progression of the hemorrhage model increases more evidently than that for the progression of the ischemia model, and the RSED oscillation (Fig. 4b) caused by blood injection (50.325 ± 4.045 dB) is stronger than that caused by the ligation of vessels (37.689 ± 1.922 dB).

### Stroke diagnosis result with the multichannel measurement

**Figure 5:**
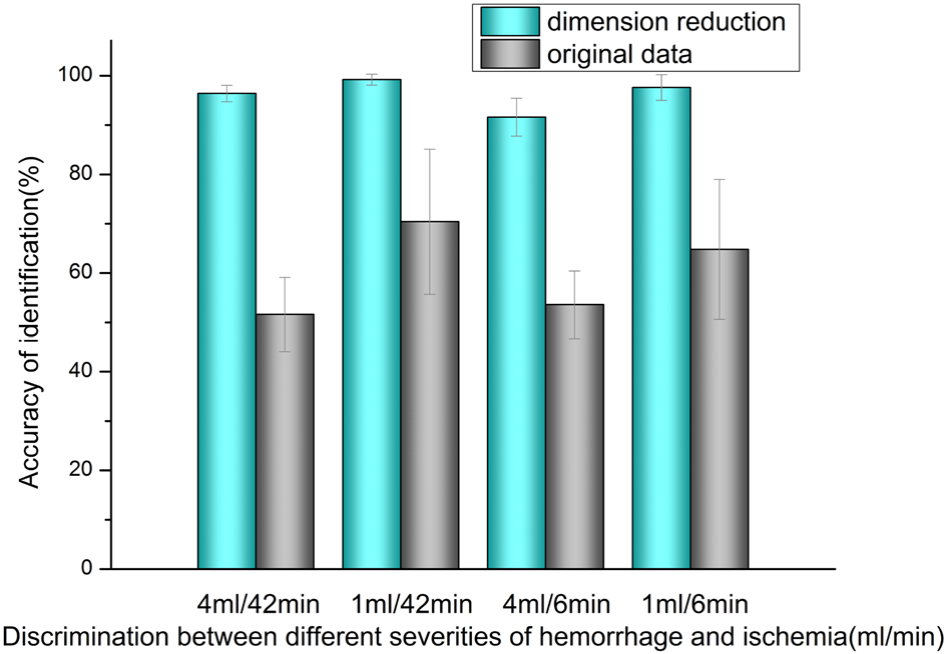
Comparison of the accuracy of stroke type recognition before and after dimensionality reduction.

The PCA dimension reduction process is implemented before classification of multi-channel RSED parameter. In contrast to the multichannel data, the single-channel data can be directly classified without the dimension reduction. Figure 5 shows the results of the identification of stroke types of different severities with and without the reduction of dimensions in data processing. In Fig. 5, the x-axis denotes the discrimination between different severities of hemorrhage and ischemia, and the y-axis denotes the accuracy of discrimination. The blue and the gray cylinders indicate the classification executed with and without the dimension reduction, respectively, in data preprocessing. The accuracy of the identification retrieved by the original data is between 50 and 70% and increases to 90–100% with dimension reduction. These results demonstrate that the dimension reduction significantly improves the diagnostic accuracy.

### Classification with different kernel functions

The SVM classification uses default parameters, and only kernel functions are adjusted. So the SVM used for stroke diagnosis and monitoring has a strong dependence on the control parameters (i.e., kernel functions). The optimal kernel function for stroke type identification should be effectively selected.

**Figure 6:**
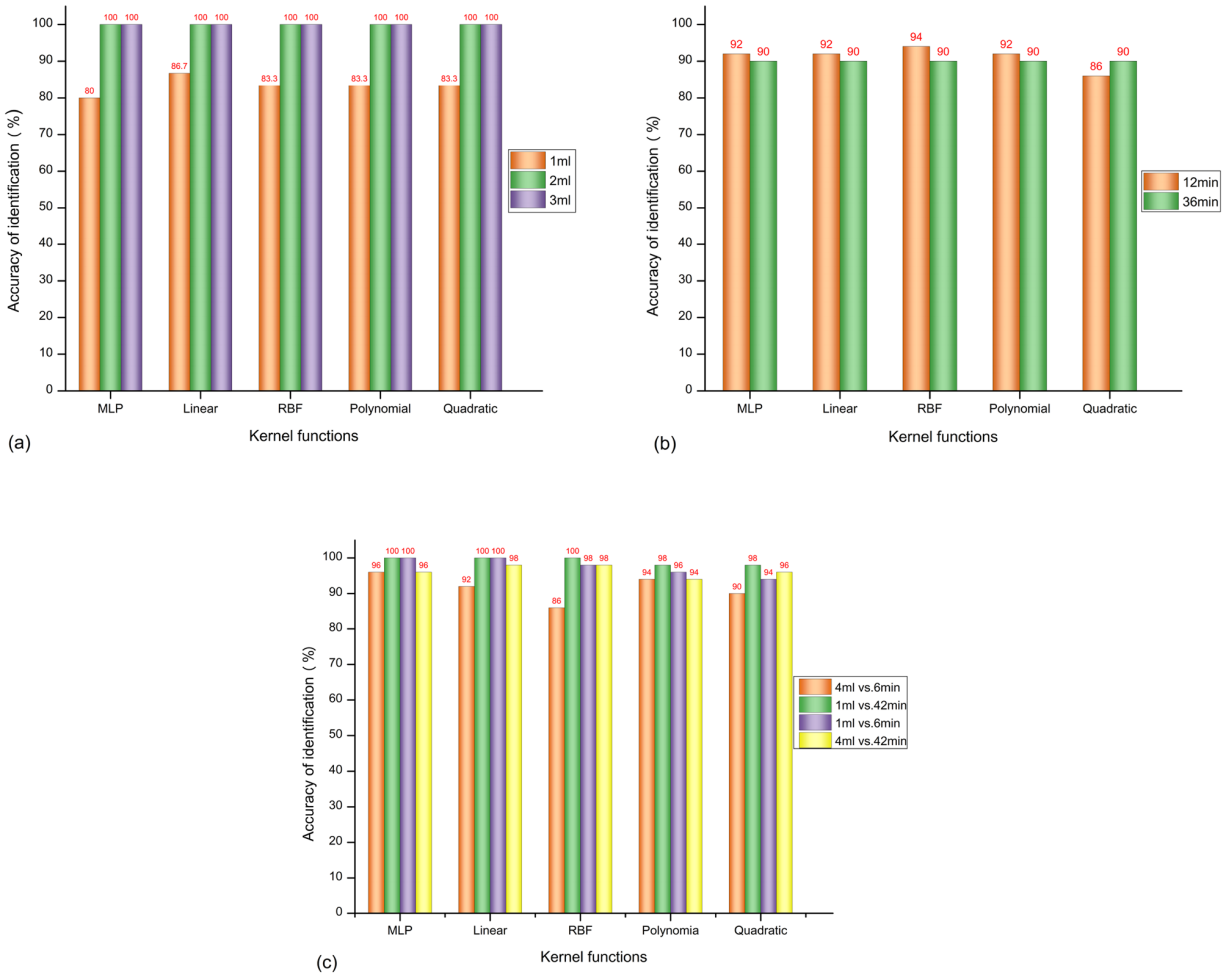
The classification results by different kernel functions: Linear kernel function, linear; multilayer perceptron kernel function, MLP; Gaussian radial basis function, RBF; quadratic kernel function, quadratic; polynomial kernels with order 3, polynomia. (**a**) The classification results of different bleeding intervals. (**b**) The classification results of different ischemic intervals. (**c**) The classification results of bleeding and ischemia.

In this section, the classification results between the hemorrhage and the ischemia models, different severities of hemorrhage, and different severities of ischemia obtained from various kernel functions are compared and shown in Fig. 6. Based on the effectiveness of the classifications performed by the various kernel functions, the multilayer perceptron (MLP) kernel is selected for the diagnosis of the stroke type (Fig. 5).

Results show that the classification accuracy reaches 100% when the amount of bleeding in the hemorrhagic stroke model is 2 and 3 ml, whereas the mean accuracy for 1 ml is only approximately 83.3%. At the same blood volume, the linear kernel function achieves the best performance with an accuracy of 86.7% (Fig. 6a).

In the cerebral ischemia experiment, the interval time of the common carotid artery ligation is set to 12 and 36 min, and different kernel functions are used to identify the ischemic severity. The mean classification accuracy is 90.6% under the various kernel functions. The Gaussian radial basis function (RBF) achieves the best result among these kernel functions with an accuracy of 94% (Fig. 6b).

Considering the differences in the pathological mechanisms of the cerebral hemorrhage and the cerebral ischemia models, the electromagnetic scattering data of the hemorrhage (maximum and minimum blood loss of 4 and 1 ml, respectively) and the ischemia (maximum and minimum common carotid artery ligation times of 42 and 6 min, respectively) models are preliminarily discriminated. The classification results are shown in Fig. 6c. The models achieve classification accuracies above 86% in the identification of hemorrhage and ischemia, and the best performance (above 96%) is achieved with the MLP.

## Discussion

In this study, the classification and the monitoring of the progression of the two stroke types are realized through a multichannel microwave transmitting–receiving detection system. The data preprocessing, calculation, feature selection, and classifier are critical factors for determining the final diagnosis accuracy of the method. Microwave electromagnetic technology is a promising method that targets the prehospital stroke diagnosis especially for patients who need timely treatment.

Currently, prehospital stroke diagnoses are based on neurological assessment scales, which are highly subjective and may fall short of accuracy^33^. The mobile CT has been adopted by some centers but is costly, nonportable, and has low temporal resolution for the prehospital triage^34^. Therefore, a noninvasive and portable device is still needed for the discrimination and the progress monitoring of stroke types. Previous reports have reported the promising identification capability of stroke types based on the microwave electromagnetic technology. In the present study, a multichannel microwave transmitting–receiving system is established.

The multichannel microwave system is verified for the diagnosis of stroke type and degree with a high accuracy (stroke type: above 96%, MLP; hemorrhagic stroke severity: above 86.7%, Linear; ischemic stroke severity: above 90%, Linear). A comparison of discrimination accuracy between our results and the previous work^31^ cannot be carried out because of the difference between experimental subjects and animal models. Clinical experiments suffer from complex pathological conditions, whereas the present experiment is conducted using animal models under controlled conditions to obtain the two stroke types.

However, the experiments demonstrate that the identification of stroke types based on the microwave electromagnetic technology is feasible, and the diagnosis accuracy can be improved by choosing the suitable classification algorithm, scattering the parameters, and decreasing the redundancy of multichannel-detected data.

The detection algorithm of Persson et al.^31^, i.e., the matched subspace detectors, is a development of the original idea of a subspace classifier and has poor nonlinear processing ability. In this paper, the scattering parameter is classified with the SVM classification algorithm, which achieves improved nonlinear processing ability. Results show that the SVM classification algorithm can be used for the stroke type and the severity identification with high classification accuracy. Among these various kernel identification functions, the MLP kernel function achieves the best performance and can be selected as the main classification kernel function for the stroke type identification.

A new scattering parameter, RSED, which combines the amplitude and the phase information of the microwave reflection data, is calculated in the experiment to improve the discrimination sensitivity. Results show that the RSED parameter has improved the discrimination sensitivity and made the system accurate to detect the severity of bleeding and ischemia. This finding indicates that this method is viable for distinguishing between bleeding and ischemia and expected to be applied to the monitoring of stroke progression in the future. However, the RSED parameters need to be classified and identified over a wide frequency range because changes in the RSED may be consistent between the hemorrhage and the ischemia in narrow band measurements.

Our previous research^32^ has confirmed the possibility of detecting the changes in hematoma by using the microwave technique. However, the classification accuracy is less than 77%. A multichannel microwave-based system is used to improve the classification accuracy. Results show that the detection accuracy with multiple channels is higher than that with a single channel, but the data dimension reduction should be conducted before the identification to reduce redundant data, which negatively affect the accuracy of the classification. The data dimension reduction should be performed before the stroke classification. Experimental results (Fig. 5) show that the accuracy of the stroke type classification is 50% ± 13.2% before the dimension reduction and increases to 96% ± 3.7% after the dimension reduction. These results indicate that the multichannel and wide-spectrum microwave detection can provide information about the physiological changes in the brains of rabbits, but the superfluous data conversely decrease the efficiency and the accuracy of diagnosis.

Although this study shows the feasibility of stroke type diagnosis and monitoring by using a microwave-based method and has achieved desirable results, some limitations remain. First, an objective evaluation is missing. Imaging methods can be used to distinguish stroke. However, under this scenario where we are conducting real-time monitoring and early diagnosis, no corresponding gold standard exists for reference. Future animal experiments will be based on an animal model of middle cerebral artery obstruction^36^ and compared with medical imaging results. Second, this study has validated that a broad band frequency range and multichannel detection method can provide more information from the brain compared with a narrow band frequency range and a single-channel detection system. However, this study also shows severe redundancy in the data from adjacent frequencies and channels. This finding can provide guidance for the design of the antenna array to select optimal frequency bands and channels to avoid severe redundancy. Third, the consistency between animal stroke models and clinical stroke needs to be improved especially the bilateral ligation ischemic stroke model, which simulates an uncommon ischemia stroke in clinics. Furthermore, the result indicates that advanced data processing and pattern recognition methods are required. Finally, state-of-the-art classification algorithms, such as deep learning, are not tested in the experiments due to the small sample size.

The developed microwave-based method shows promise in meeting the urgent need to promptly diagnose and monitor stroke. In future clinical trials, some new sensors^16,21,35^ will be used to optimize the system and a new construction of antenna array that can minimize the data redundancy. At the same time, new classification algorithms and specific frequency band suitable for human diagnosis will be evaluated to improve the diagnostic results. Also, for the optimal classification in different clinical situations, a factor will be considered to regulate the detected system to obtain a balance between sensitivity and specificity. The real-time capability of the detection system should also be considered in future work.

## Materials and methods

### Microwave detection principle

The theoretical basis for the microwave scattering parameter stroke detection is based on the different dielectric properties of different tissues in the brain^37,38^. The dielectric properties of the blood and the cerebrospinal fluid (CSF, or edema) are vastly different from those of white and gray matter (Table 1)^39^. Under pathological conditions, these changes in dielectric properties cause changes in the microwave scattering parameters.

**Table 1:** Permittivity and conductivity of brain tissue at 1.2 GHz.

Tissue	Permittivity	Conductivity (s/m)
Cerebrospinal fluid	68	2.4552
Gray matter	52	0.98541
White matter	46	0.82431
Blood	61	1.5829

In the early stage of cerebral hemorrhage, a large amount of bleeding causes acute swelling damage to the tissue, and the CSF is first excluded from the cranial cavity^40^. Generally, the hematoma forms after 20–30 min of hemorrhage. The enlargement of the hematoma causes elevated intracranial pressure, compresses the brain tissue, causes blood flow disorders, forms ischemia, and produces edema^41^. Thus, the change in the conductivity of the whole brain during the early stage of cerebral hemorrhage can be affected by the volume of cerebral hemorrhage, cerebral blood flow, and CSF.

In the process of cerebral ischemia, the cerebral blood flow decreases, and the sodium and the potassium pumps in the cell membrane stop working when the brain cells are deprived of oxygen. The difference in ion concentrations between the inside and the outside of the membrane causes an accumulation of sodium ions in the cell. With the accumulation of sodium ions, the outflow of potassium ions, continued rise of osmotic pressure, entrance of water molecules to the cells, and swelling of brain cells are observed, causing an edema^42,43^. The ischemia leads to increased local carbon dioxide and vasodilatation, which also causes the cerebral edema^44^. Therefore, the main factor leading to changes in the intracranial impedance is the brain cell edema in the early stage of cerebral ischemia.

**Figure 7:**
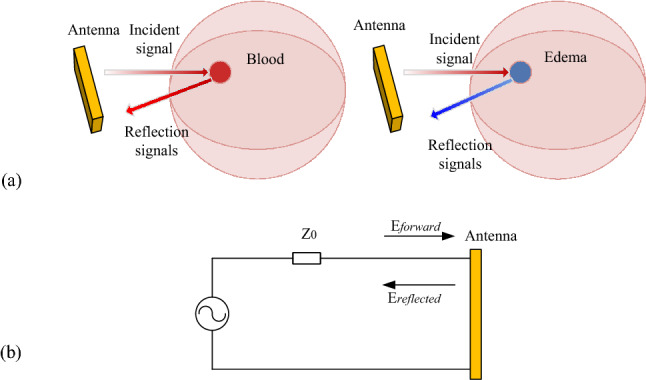
(**a**) Reflection pattern detection principle of microwave; (**b**) transmission-line theory model of microwave.

Different mechanisms in the early stages of the two types of stroke can cause different changes in the dielectric properties of the brain. The different dielectric properties of these pathological tissues produce different microwave reflection patterns (Fig. 7a), providing a theoretical basis for using microwave scattering parameters to identify stroke types.

The microwave scattering parameters (or S parameters) originate from the transmission line theory and are defined in terms of the transmitted and the reflected voltage waves. A previous study^29^ has proven that the scattering parameters can be used for stroke detection, which reflect the changes in the dielectric properties of different cerebral tissues. In the present study, the scattering parameters are transformed using the Euclidean distance transformation to highlight the differences in dielectric properties caused by ischemia and hemorrhage. The Euclidean distance transformation that reflects the amplitude and the phase information of the scattering parameters is calculated as follows.

Figure 7b shows a voltage source feeding into the antenna with a source impedance of *Z*_0_. $$ \mathop a\limits^{ \rightharpoonup }  = \frac{{\overset{\lower0.5em\hbox{$\smash{\scriptscriptstyle\rightharpoonup}$}}{{E_{{{\text{forward}}}} }} }}{{\sqrt {\overset{\lower0.5em\hbox{$\smash{\scriptscriptstyle\rightharpoonup}$}}{{z_{0} }} } }} $$ and $$\mathop{b}\limits^{\rightharpoonup} = \frac{{\overset{\lower0.5em\hbox{$\smash{\scriptscriptstyle\rightharpoonup}$}}{{E_{{{\text{reflected}}}} }} }}{{\sqrt {\overset{\lower0.5em\hbox{$\smash{\scriptscriptstyle\rightharpoonup}$}}{{z_{0} }} } }}$$ are defined as the square root of the forward and the reflected incident microwaves, respectively^29^. The scattering function can be expressed as the vector difference of $$\mathop{a}\limits^{\rightharpoonup} $$ and $$\mathop{b}\limits^{\rightharpoonup} $$:

1 $$ \mathop{k}\limits^{\rightharpoonup} = \frac{{\mathop{b}\limits^{\rightharpoonup} }}{{\mathop{a}\limits^{\rightharpoonup} }} = \frac{{\overset{\lower0.5em\hbox{$\smash{\scriptscriptstyle\rightharpoonup}$}}{{E_{{{\text{reflected}}}} }} }}{{\overset{\lower0.5em\hbox{$\smash{\scriptscriptstyle\rightharpoonup}$}}{{E_{{{\text{forward}}}} }} }} $$

2 $$ \beta = {\text{abs}}(\mathop{k}\limits^{\rightharpoonup} ),\quad {\text{and}}\quad \theta = {\text{phase}}(\mathop{k}\limits^{\rightharpoonup} ) $$

$\beta$ is the magnitude value, and $\theta$ is the phase value of vector $\mathop{k}\limits^{\rightharpoonup}$.

For the cerebral hemorrhage, the magnitude values of 0 (baseline) and *n* ml of hemorrhages are expressed as $\beta_{0}$ and $\beta_{n}$, respectively, and the phase values of 0 (baseline) and *n* ml of hemorrhages are expressed as $\theta_{0}$ and $\theta_{n}$, respectively.

The two magnitude values are transformed into the exponential form:

3 $$ \beta_{0} ^{\prime} = 10^{{\frac{{\beta_{0} }}{20}}} ,\quad {\text{and}}\quad \beta_{n} ^{\prime} = 10^{{\frac{{\beta_{n} }}{20}}} $$

The modulus of vector $\mathop{k}\limits^{\rightharpoonup}$ of *n* ml hemorrhage is calculated on the basis of the magnitude exponential form and phase.

4 $$ \varepsilon_{n} = \beta_{0} ^{{\prime}{2}} + \beta_{{\text{n}}} ^{{\prime}{2}} - 2\beta_{0} ^{\prime}\beta_{{\text{n}}} ^{\prime}\cos (\theta_{n} - \theta_{0} ) $$

The logarithmic transformation is performed on the obtained modulus value of the scattering parameter of *n* ml of hemorrhage, which is defined as the RSED of *n* ml of hemorrhage:

5 $$ {\text{RSED}}_{{\text{n}}} = 20{\text{l}} {\text{g}}\varepsilon_{n} $$

The calculation of the RSED for cerebral ischemia is similar to that for the cerebral hemorrhage, except that the *n* is the duration of the ischemia (minutes).

By introducing the RSED, the magnitude and the phase of the microwave scattering parameters are merged into one variable to differentiate the hemorrhagic and the ischemic stroke. The transformation by RSED restrains the fast changes in the original data caused by the external disturbance through the logarithmic conversion process.

### Detection system

**Figure 8:**
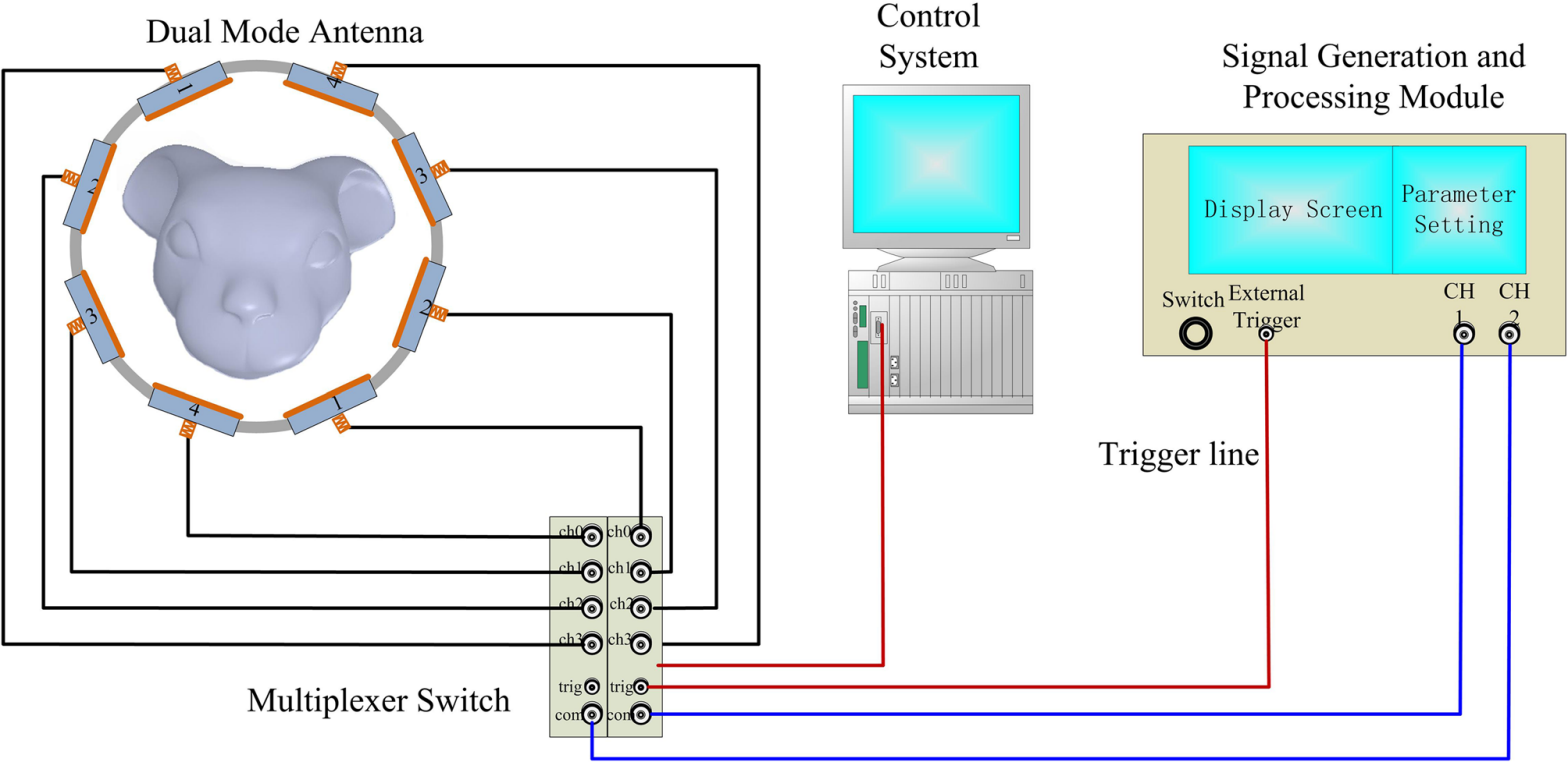
Construction of the multichannel microwave-based monitor system.

A multichannel microwave-based monitoring system is built for stroke detection to detect the spatial dielectric properties of the whole brain. The system is composed of an antenna array sensor (8 antennae), a microwave monitoring device (KEYSIGHT E5061B), and a multiplexer switch (PXI-2544) controlled by the PXI mainframe (PXIe-8840) and connected as shown in Fig. 8.

The microwave-based monitor device provides the incident microwave signal and processes the receiving signal. The antenna array sensor is used to transmit and receive microwaves and acquire physiological and pathological information from the brain. The multiplexer switch is used to sequentially connect the transmitting and the receiving signals from the microwave-based monitor device to the dual-mode antenna one pair at a time because the number of antenna array channels is larger than the number of ports on the microwave-based monitor device. Given that the repetition period of each signal is short, the multichannel signals can be measured simultaneously.

The transmitted microwave power of the microwave-based monitor device is 10 dBm, and the measurement frequency ranges from 300 kHz to 3 GHz (in 3 MHz steps)^32^. The intermediate frequency bandwidth is 30 kHz.

#### Antenna array sensor

The antenna array sensor is composed of eight microwave patch antennae^32^ (4 receivers and 4 transmitters) that are fixed onto an acrylic loop, as shown in Fig. 8. The multiplexer switch controls 16 channels (4 receivers × 4 transmitters) in turn to launch the microwave monitor device. The head of the rabbit is placed in the center of the sensor array during the experiment, and the active antenna emits microwaves through the head.

#### Multichannel detection implementation

Sixteen channels, each at different orientations, are switched by the multiplexer switch. The multichannel detection is realized through the time division multiplexing to avoid interference among different detection channels. After the one-channel measurement is completed, the multiplexer switches quickly and prepares for the next channel detection. The specific implementation is described as follows. (1) On the microwave monitoring device, the data measurement and recording programs for signal detection are written using the Visual Basic for Applications, which waits for an external trigger signal after starting. (2) The PXI mainframe, into which the multiplexer switch is embedded, controls the on-state of the two 4-channel multiplexers to traverse all time-sharing detection channels. (3) When the multiplexer switch is turned on for one channel, the PXI mainframe sends an external trigger signal to the microwave monitoring device, sets a short delay to wait for the system to complete the measurement, and saves the data from this channel.

### Animal experiments

All animal experiments are performed in accordance with the guidelines from the Administration of Animal Experiments for Medical Research Purposes issued by the Ministry of Health of China. The protocol used is reviewed and approved by the Animal Experiments and Ethical Committee of Third Military Medical University (Chongqing, China). Ten rabbits (available from the Laboratory Animal Center of the Third Military Medical University, weighing 2.0–2.6 kg) are divided into cerebral hemorrhage (marked No. 1 to No. 5) and cerebral ischemia (marked No. 6 to No. 10) groups. All rabbits are first injected with urethane (25%, 5 ml) in an ear vein for anesthesia.

#### Internal capsule cerebral hemorrhage model for the cerebral hemorrhage group

The internal capsule cerebral hemorrhage model with autologous blood injection^32,45–47^ is used for the cerebral hemorrhage group. The hair and the scalp of the rabbits are removed from the head, and a hole (diameter = 1 mm) is drilled beside the bregma, 6 mm to the right of the coronal suture, and 1 mm posterior to the sagittal suture. A plastic fiber tube (diameter = 1 mm) is connected to the injection pump and quickly punctured at this point to a perpendicular depth of 13 mm. The hole is sealed with dental cement. As shown in Fig. 1, the rabbits are placed into the antenna array sensor after the above operation. The data are measured before the blood injection as a baseline (0 ml). Then, 1 ml autologous blood collected from the femoral artery is injected with an electronic injection pump (long pump) at a rate of 1 ml per minute, and the microwave data are recorded and stored. The steps are repeated to complete data acquisition at 2, 3, and 4 ml.

#### Bilateral carotid artery ligation model for the cerebral ischemic group

**Figure 9:**
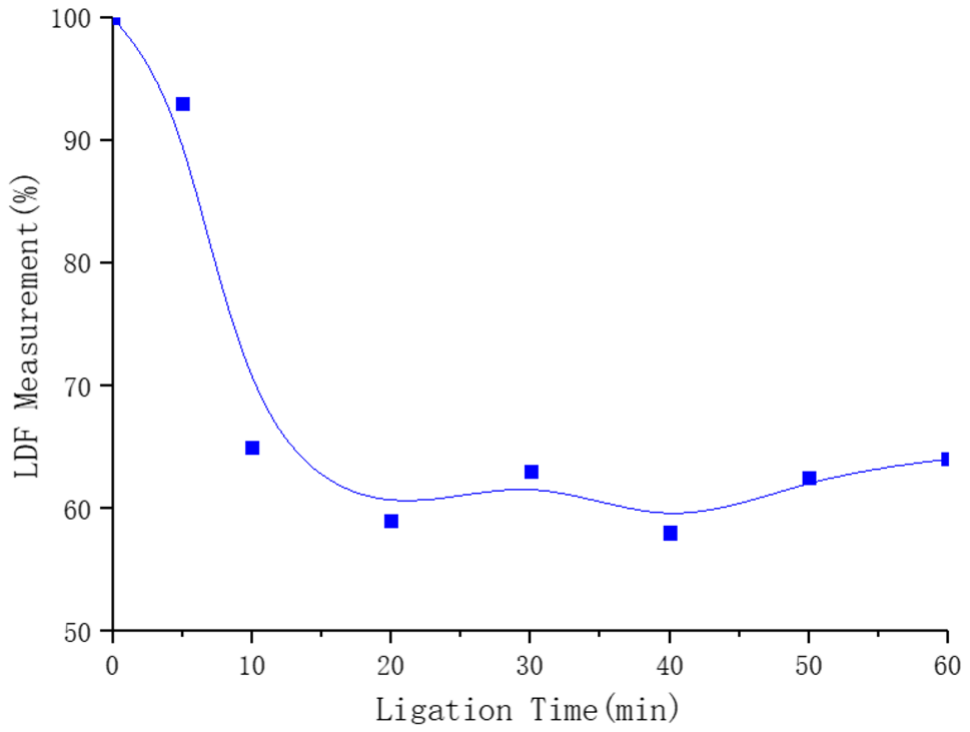
The cerebral blood changes after ligation of the bilateral common carotid artery.

The rabbit global cerebral ischemia model is prepared using the bilateral common carotid artery ligation method^48,49^. The brain blood flow after ligation is measured using the Periflux System F5001 (PERIMED AB, Järfäll, Sweden). The brain blood flow rapidly declines within 10 min after stabilization at a relatively low level (Fig. 9), thereby showing the successful establishment of the cerebral ischemia model.

The rabbits are depilated at the neck. The bilateral common carotid artery is exposed and separated, and a surgical suture is placed beneath the artery. The rabbit is then placed in a fixed detection position within the sensor array. After measuring the baseline (0 min), the bilateral common carotid arteries are ligated with the line under the artery. The multiplexer switch is set to automatically perform one measurement every 6 min. The measurement is repeated six times for a total recording time of 42 min.

### Signal processing and statistical analysis

#### Stroke classification

The classification of stroke based on the multichannel system is carried out through the following steps. (1) The microwave scattering parameters of the whole brain of rabbits are collected after the stroke operation. Bleeding and ischemic rabbits are monitored for 5 and 42 min, respectively. (2) The scattering parameter to the RSED parameter, which serves as the input parameter of the classifier, is reconstructed (Eqs. 1–5). The control group data of 0 ml or 0 min are utilized as the baseline data to calculate the RSED parameter. (3) Data are pre-processed before classification (see “Data pre-processing” section). Unlike the single-channel data, the multichannel data require the dimension reduction. (4) The training and the classification of the pattern recognition classifier are conducted on the basis of pre-processed data. The classification algorithm of the SVM is selected for the stroke classification (see “Classification algorithm” section). (5) The type of stroke is judged using the classifier. Finally, the statistical analysis of the RSED parameter, classification results of the dimension reduction, and different kernel functions is performed. The classification of stroke based on the single-channel system also follows the above steps but without the dimension reduction. The details of the data pre-processing, classification algorithm, and statistical analysis are shown below.

#### Data pre-processing

Before statistical analysis, the RSED is filtered and smoothed by a moving average to produce the data to be classified. All data preprocessing methods are implemented in the Matlab R2014a (The MathWorks, Inc., USA).

Redundant information in the data is observed from the multichannel measurements. These redundant data affect the speed and the accuracy of classification. Therefore, before using the multidimensional data for stroke classification, the dimensionality of the raw data should be reduced to remove the redundancy. This paper uses the principal component analysis (PCA) for data reduction.

The PCA retains low-order principal components, reduces high-order principal components to achieve dimension reduction, and maintains the maximum contribution to the variance by the projection of the data. The effective retention of low-order principal components is consistent with the requirement that the dimension reduction needs to preserve the most important part of the data^50^. The PCA dimension reduction process is also implemented using the Princomp function in the Matlab R2014a.

**Figure 10:**
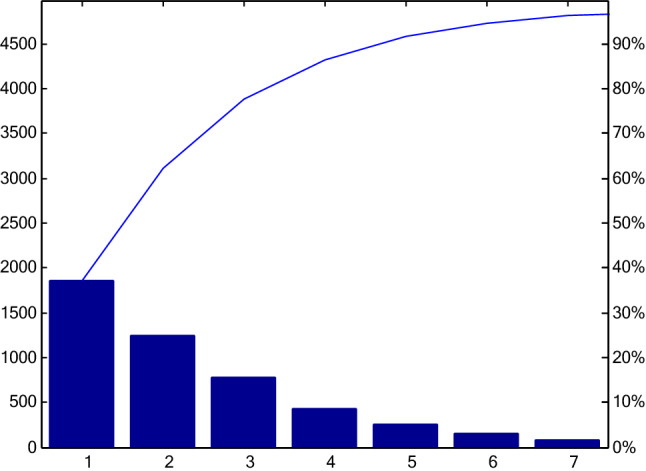
The 7-dimensional data contribution rate after principal component analysis.

Ten sets of data are enrolled in the dimension reduction for classification purpose. A total of 44 × 1001 data points are collected in the training set consisting of eight rabbits. Another two sets of data is left for validation. The PCA reduces the data dimension into 44 × 7 data points, i.e., 7-dimensional data. The 7-dimensional data retains more than 95% of the original data (Fig. 10), indicating most dramatic changes in electromagnetic parameters caused by bleeding or ischemia. In addition, the dimension reduction reduces the invalid data that weaken the detection of bleeding or ischemia, which improves the detection performance.

#### Classification algorithm

According to the machine learning theory, the statistical learning theory is suitable for small sample cases. Therefore, the SVM classification algorithm based on statistical learning is chosen. The SVM is a common method of pattern recognition and automatically adjusts the model structure by controlling the parameters to minimize the classification of the empirical and the structural risks. This paper uses the LIBSVM toolkit provided by Chih-Chung Chang and Chih-Jen Lin^51,52^ to implement the classification algorithm on RSED. The Svmtrain and the Svmclassify functions are used to train and validate the SVM classifier, and the Five-Fold Cross Validation is used for validation. The severity of bleeding is taken as an example. Four of the five bleeding rabbits are used for training, and one rabbit is used for verification. The average of all the same stroke states is obtained. For example, the detection performance of the 1 ml interval of cerebral hemorrhage is obtained by averaging the results of the classification of 1 ml versus 2 ml, 2 ml versus 3 ml, and 3 ml versus 4 ml. Another example is stroke type classification. The 10 rabbits are divided into 5 groups with 2 rabbits in each group, of which 4 groups are used as training sets and the remaining 1 group is used as test sets. Five tests are conducted, and the average classification accuracy is obtained from the results of the five classification tests.

The RSED is directly classified using the SVM without extracting features. The SVM classification uses default parameters, and only kernel functions are adjusted. Considering the linear and the nonlinear classification, aside from the linear kernel function, some other kernel functions are adjusted to achieve optimal classification peformance^53^. The classification results from implementing linear, MLP, a Gaussian RBF, quadratic, and polynomial kernels with order 3 are compared. SVM performers as follow parameter setting: RBF, the scaling factor, sigma, in the radial basis function kernel is 1; polynomial, specifies the order of a polynomial kernel is 3; Multilayer Perceptron kernel (MLP) was selected with weight 1 and bias − 1; Sequential Minimal Optimization (SMO) method was used to find the separating hyperplane; Value of the box constraint C for the soft margin was set to be default in experiments.

### Statistical analysis

The RSED of the single-channel measurements is calculated for the two groups of rabbits and expressed as the mean ± SD. The results for the different intervals (hemorrhagic stroke: volume, ischemic stroke: duration) are shown in Fig. 2. The multichannel RSED of the hemorrhagic and the ischemic rabbits is analyzed and expressed as the mean values of the 16-channel measurement results (Fig. 4).

The two models of stroke are caused by different mechanisms. Thus, the level of bleeding is quantified using the volume, and the severity of the ischemia is quantified using the time. Therefore, for the statistical analysis of the discrimination results, the minimum (bleeding: 1 ml, ischemia: 6 min) and the maximum (bleeding: 4 ml, ischemia: 42 min) intervals for the two types of stroke are used to compare the discrimination accuracy. Results are shown in the histograms in Figs. 3, 5, and 6. Statistical analyses are performed using the Origin software version 2017 (Originlab Inc., Northampton, USA).
